# LKB1 and AMPK differentially regulate pancreatic β-cell identity

**DOI:** 10.1096/fj.14-257667

**Published:** 2014-11

**Authors:** Marina Kone, Timothy J. Pullen, Gao Sun, Mark Ibberson, Aida Martinez-Sanchez, Sophie Sayers, Marie-Sophie Nguyen-Tu, Chase Kantor, Avital Swisa, Yuval Dor, Tracy Gorman, Jorge Ferrer, Bernard Thorens, Frank Reimann, Fiona Gribble, James A. McGinty, Lingling Chen, Paul M. French, Fabian Birzele, Tobias Hildebrandt, Ingo Uphues, Guy A. Rutter

**Affiliations:** * Section of Cell Biology and; † Section of β-Cell Development, Division of Diabetes, Endocrinology, and Metabolism, Department of Medicine, and; ‡ Photonics Group, Department of Physics, Imperial College London, London, UK;; § Swiss Institute of Bioinformatics and; ‖ Center for Integrative Genomics, University of Lausanne, Lausanne, Switzerland;; ¶ Department of Developmental Biology and Cancer Research, Institute for Medical Research Israel–Canada, Hebrew University–Hadassah Medical School, Jerusalem, Israel;; # AstraZeneca Diabetes and Obesity Drug Discovery, Alderley Edge, UK;; ** Metabolic Research Laboratories, University of Cambridge, Cambridge, UK and; †† Boehringer Ingelheim Pharma, Ingelheim, Germany

**Keywords:** islet, diabetes, insulin secretion, RNASeq

## Abstract

Fully differentiated pancreatic β cells are essential for normal glucose homeostasis in mammals. Dedifferentiation of these cells has been suggested to occur in type 2 diabetes, impairing insulin production. Since chronic fuel excess (“glucotoxicity”) is implicated in this process, we sought here to identify the potential roles in β-cell identity of the tumor suppressor liver kinase B1 (LKB1/STK11) and the downstream fuel-sensitive kinase, AMP-activated protein kinase (AMPK). Highly β-cell-restricted deletion of each kinase in mice, using an *Ins1*-controlled *Cre*, was therefore followed by physiological, morphometric, and massive parallel sequencing analysis. Loss of LKB1 strikingly (2.0–12-fold, *E*<0.01) increased the expression of subsets of hepatic (*Alb, Iyd, Elovl2*) and neuronal (*Nptx2, Dlgap2, Cartpt, Pdyn*) genes, enhancing glutamate signaling. These changes were partially recapitulated by the loss of AMPK, which also up-regulated β-cell “disallowed” genes (*Slc16a1, Ldha, Mgst1, Pdgfra*) 1.8- to 3.4-fold (*E*<0.01). Correspondingly, targeted promoters were enriched for neuronal (Zfp206; *P*=1.3×10^−33^) and hypoxia-regulated (HIF1; *P*=2.5×10^−16^) transcription factors. In summary, LKB1 and AMPK, through only partly overlapping mechanisms, maintain β-cell identity by suppressing alternate pathways leading to neuronal, hepatic, and other characteristics. Selective targeting of these enzymes may provide a new approach to maintaining β-cell function in some forms of diabetes.—Kone, M., Pullen, T. J., Sun, G., Ibberson, M., Martinez-Sanchez, A., Sayers, S., Nguyen-Tu, M.-S., Kantor, C., Swisa, A., Dor, Y., Gorman, T., Ferrer, J., Thorens, B., Reimann, F., Gribble, F., McGinty, J. A., Chen, L., French, P. M., Birzele, F., Hildebrandt, T., Uphues, I., Rutter, G. A. LKB1 and AMPK differentially regulate pancreatic β-cell identity.

Diabetes mellitus is a socioeconomically costly disease affecting ∼8% of the adult population worldwide (1). The most prevalent form, type 2 diabetes (T2D), involves a decline in the number of normally functioning β cells through incompletely defined mechanisms (2).

Glucose-induced insulin secretion from the healthy pancreatic β cell requires intracellular metabolism of the sugar, increased intracellular ATP/ADP levels, and elevated free Ca^2+^ (3). Although T2D progression in humans is characterized by a limited decrease in overall β-cell mass (4, 5) it is increasingly apparent that a loss of the normal differentiated state of the remaining cells, and their consequent “glucose blindness,” also plays a role (6–9). As well as decreased expression of signature genes, such as the glucose transporter Glut2/*Slc2a2* and glucokinase (*Gck*) (6), β-cell dedifferentiation is characterized by the increased expression of normally repressed (“disallowed”) genes, such as *LDHA* and the lactate transporter *MCT-1/Slc16a1* (10), leading to aberrant fuel sensing (11).

Liver kinase B1 (LKB1), also called STK11, is a mammalian Ser/Thr kinase and tumor suppressor whose invertebrate homologue (Par-4; ref. 12) controls embryo polarity. LKB1 was shown [alongside calmodulin kinase kinase 2 and transforming growth factor (TGF)-β-activated kinase (TAK); ref. 13] to be one of 3 physiologically relevant upstream kinases for AMP-activated protein kinase (AMPK; refs. 14, 15), and other members of the AMPK-related kinase (AMPKRK) family (16), previously implicated in β-cell glucose sensing (17, 18). Demonstrating the role of LKB1 in restricting cell growth in humans, mutations in the human *LKB1* gene lead to Peutz-Jegers syndrome (19), an autosomal dominant disorder characterized by the development of intestinal polyps.

We (20, 21) and others (22) have previously demonstrated that inactivation of either LKB1 or AMPK (23, 24) selectively in pancreatic β cells and a small number of other cell types exerts dramatic effects on insulin secretion *in vivo*. Thus, loss of LKB1 causes β-cell hyperplasia and an increase in overall β-cell mass, associated with dramatic changes in cell polarity. In marked contrast, deletion of both AMPK catalytic subunits (α1, global; and α2, β cells, brain) had no effect on β-cell size and mass but strongly inhibited insulin secretion *in vivo* (23, 24). The molecular underpinnings of these changes remain, however, unexplored.

To examine in detail the cell autonomous roles of LKB1 and AMPK in the β cell, we have therefore developed new models using recombination based on *Cre* expression under *Ins1* promoter control, avoiding deletion in the brain (25, 26). Metabolic analysis and massive parallel sequencing of islets from each model reveal both overlapping and distinct roles for LKB1 and AMPK in β cells. We show that these enzymes are essential to avoid the misexpression of a subset of genes normally expressed at relatively low levels in β cells, including those involved in glutamate signaling and in allowing alternative metabolic fates for glucose.

## MATERIALS AND METHODS

### Generation of mutant mice lacking LKB1 selectively in pancreatic β cells

Mice heterozygous for floxed alleles of the *Lkb1/Stk11* gene (mixed FVB/129S6 and C57BL/6 background; ref. 27) were obtained from the Mouse Models of Human Cancer Consortium [U.S. National Institutes of Health (NIH), Bethesda, MD, USA; http://www.nih.gov/science/models/mouse/resources/hcc.html] and backcrossed with C57BL/6 mice 4 times. Mice were then crossed with mice expressing *Cre* under the mouse *Ins1* promoter (Ins1.Cre), and the resulting heterozygous mice were intercrossed with siblings to generate Ins1LKB1-knockout (Ins1LKB1KO) mice (*Lkb1*^*fl/fl*^, *Cre*^+^). Ins1LKB1KO mice were further bred with *Lkb1*^*fl/fl*^ mice to generate littermate controls (*Lkb1*^*fl/fl*^).

### Generation of mutant mice selectively lacking AMPK α1 and α2 in pancreatic β cells

Mice homozygous for *Ampk*α*1*^*fl/fl*^ (Dr. Benoit Viollet, Institut National de la Santé et de la Recherche Médicale, U1016, Paris, France) were crossed to mice heterozygous for floxed alleles of AMPKα2 (*Ampk*α*2*^*fl*/+^; ref. 23). The resulting double heterozygotes (*Ampk*α*1*^*fl*/+^, α*2*^*fl*/+^) were crossed with Ins1Cre-expressing animals to generate triple heterozygous mice (*Ampk*α*1*^*fl*/+^, α*2*^*fl*/+^, *Cre*^+^). The latter were then bred with mice homozygous for both floxed *Ampk*α*1* and α*2* alleles (*Ampk*α*1*^*fl/fl*^, α*2*^*fl/fl*^) to produce Ins1AMPK double-KO (Ins1AMPKdKO) mice (*Ampk*α*1*^*fl/fl*^, α*2*^*fl/fl*^, *Cre*^+^). Ins1AMPKdKO mice were further crossed with *Ampk*α*1*^*fl/fl*^, α*2*^*fl/fl*^ mice to generate littermate controls (*Ampk*α*1*^*fl/fl*^, α*2*^*fl/fl*^). All mice were maintained on a C57BL/6 background.

### Mouse maintenance and diet

Animals were housed 2 to 5 per individually ventilated cage in a pathogen-free facility with 12-h light-dark cycle and had free access to standard mouse chow diet. All *in vivo* procedures described were performed at the Imperial College Central Biomedical Service and approved by the UK Home Office Animals Scientific Procedures Act, 1986 (HO License PPL 70/7349).

### Isolation of mouse islets and β cells

Islets were isolated by pancreatic distension and digestion with collagenase as described previously (28). β Cells were purified by fluorescence-activated cell sorting (FACS) as described previously (29) and directly collected in Trizol (Life Techonologies, Grand Island, NY, USA).

### RNA extraction and massive parallel RNA sequencing (RNAseq)

Islets (50–100) extracted from Ins1LKB1KO or Ins1AMPKdKO mice and their wild-type (WT) controls, age 12–14 wk, were incubated in RPMI medium containing 11 mM glucose, 10% FCS, 100 IU/ml penicillin, and 100 μg/ml streptomycin, at 5% CO_2_ and 37°C for 24 h prior to being lyzed in RNA lysis buffer using the RNAeasy kit according to the manufacturer's instructions (Qiagen, Valencia, CA, USA).

### Library preparation and sequencing

All libraries were prepared using the TruSeq RNA Sample Preparation Kit v2 (Illumina, San Diego, CA, USA) according to the manufacturer's instructions. In brief, magnetic beads containing polydT molecules were first used to purify mRNA from 250 ng of total RNA. Second, samples were chemically fragmented and reverse transcribed into cDNA. Finally, end repair and A-base tailing was performed before Illumina adapters were ligated to the cDNA fragments. Purified samples were amplified by 15-cycle PCR. Amplified material was validated and quantified using an Agilent 2100 bioanalyzer and the DNA 1000 Nano Chip Kit (Agilent, Technologies, Santa Clara, CA, USA).

Libraries were loaded onto the channels of the flowcell at 9 pM concentration. Sequencing was carried out on the Hiseq 2000 (Illumina) by using Illumina's Trueseq Single Read Cluster Generation Kit v3 CBot Hs and running 50 cycles with the Cycle Sequencing Kit according to the manufacturer's instructions.

### Transcriptomic data analysis and identification of putative transcription factor binding sites

Datasets from RIP*Cre* strains had reads mapped to the mouse genome (Ensembl56) using the Genomatix Mapping station algorithm (allowing for up to 3 mismatches, no indels; Genomatix, Munich, Germany). Reads were additionally mapped to a set of (artificial) splice junctions of all known exons in the mouse preserving exon order within a gene. Reads from Ins1*Cre* strains were mapped to the mouse genome (Ensembl66) using the Bowtie2-Tophat2 spliced read mapper (30). Differential expression was analyzed using SageBetaBin (31), and expression values [reads per kilobase of transcript per million reads read (RPKM)] were calculated according to Mortazavi *et al.* (32). Transcription factor binding sites (TFBSs) enriched in the promoters of differentially expressed genes were identified using the Whole Genome rVista tool (33).

Kyoto Encyclopedia of Genes and Genomes (KEGG) pathway analysis (Kanehisa Laboratories, Kyoto, Japan; http://www.genome.jp/kegg/) was performed using the Database for Annotation, Visualization and Integrated Discovery (DAVID) functional analysis tool (U.S. National Institute for Allergy and Infectious Diseases, NIH, Bethesda, MD, USA; http://david.abcc.ncifcrf.gov). Expression patterns in mouse tissues and the likely functions of identified genes were assessed by reference to BioGPS (Scripps Research Institute, La Jolla, CA, USA; http://biogps.org/).

### Gene set enrichment analysis (GSEA)

GSEA (34) was performed by first ranking differentially expressed genes according to fold change (high to low; either absolute or taking direction into account) and testing for enrichment against MSigDB V4 gene sets, or a gene set containing disallowed genes. Empirical *P* values were calculated by performing a bootstrap where gene labels were shuffled 10,000 times and the enrichment was recalculated. An estimate of the false discovery rate (FDR) was calculated using the Benjamini-Hochberg procedure using the Bioconductor *multtest* package in R (35). An FDR of 30% was considered as indicating significant enrichment.

### RNA-Seq accession codes

Raw sequence data for RNA-Seq are available at the European Molecular Biology Laboratory–European Bioinformatics Institute (EMBL-EBI) ArrayExpress website (accession number E-MTAB-2791; https://www.ebi.ac.uk/arrayexpress/).

### Imaging of pancreatic slices and optical projection tomography (OPT)

Permeabilized slices were prepared as described previously (20) and blotted with primary antibodies, anti-guinea pig insulin (1:200; Dako, Ely, UK), anti-rabbit glucagon (GCG; 1:100; Santa Cruz Biotechnology, Santa Cruz, CA, USA), and anti-rabbit NPTX2 (5 μg/ml; Abcam, Cambridge, UK). Slices were visualized with Alexa Fluor 488 goat anti-guinea pig IgG and with Alexa Fluor 568 donkey anti-rabbit IgG (Invitrogen, Paisley, UK) using an Axiovert 200 M microscope (Zeiss, Welwyn Garden City, UK). ImageJ software (Wayne Rasband, NIH, Bethesda, MD, USA) was used to calculate β- and α-cell mass. OPT of whole pancreata stained with anti-insulin antibodies was performed as described previously (20).

### Islet isolation for calcium imaging

Pancreatic islets were isolated as described above, dispersed into single β cells, and plated on glass coverslips (36). Imaging experiments were performed essentially as described previously (37) on an Olympus IX-71 microscope with ×40 objective, using an Andor CMOS Zyla camera (Andor Technology, Belfast, UK) and MT-20 excitation system equipped with a Hg/Xe arc lamp controlled by Micro-Manager software (38). Images were acquired at a frequency of 0.5 Hz with typical excitation times of 50 ms.

### Statistical analysis

Data are presented as means ± sem. Significance was assessed by Student's *t* test with appropriate Bonferroni correction, or ANOVA using Prism (GraphPad, San Diego, CA, USA).

## RESULTS

### Effect on glucose homeostasis and insulin secretion of deleting LKB1 or AMPK highly selectively in the β cell with Ins1*Cre*

To avoid the complications associated with deletion of LKB1 (39) or AMPK (23, 24) in the brain after Ins2*Cre*-mediated recombination (40) we used a knock-in mouse in which *Cre* recombinase was introduced at the *Ins1* locus (refs. 25, 26 and unpublished results). In contrast to RIP2LKB1KO mice, postnatal body weight gain was normal in Ins1LKB1KO animals; for males aged 10–14 wk: WT, 26.9 ± 0.4 g (*n*=4 mice), and for Ins1LKB1KO, 27.4 ± 0.9 g (*n*=8, *P*>0.05). Furthermore, Ins1LKB1KO animals showed no signs of paralysis or premature mortality up to 12 mo of age (results not shown).

**Figure 1.**
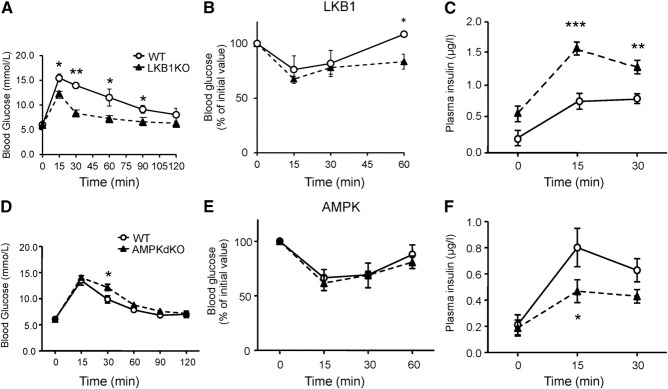
Glucose homeostasis and insulin secretion in Ins1LKB1KO and Ins1AMPKdKO mice. *A–C*) Glucose (1 g/kg intraperitoneal) tolerance (*A*), insulin (0.75 U/kg) tolerance (*B*), and glucose (3 g/kg)-induced insulin secretion (*C*) in WT or Ins1LKB1KO mice (10- to 12-wk-old males, *n*=3 WT, 5 KO). *D–F*) As in *A–C* but comparing WT (AMPKα1^f/f^:AMPKα2^f/f^) and Ins1AMPKdKO mice (*n*=5–10 mice/genotype). *P < 0.05, **P < 0.01 for effects of genotype.

**Figure 2.**
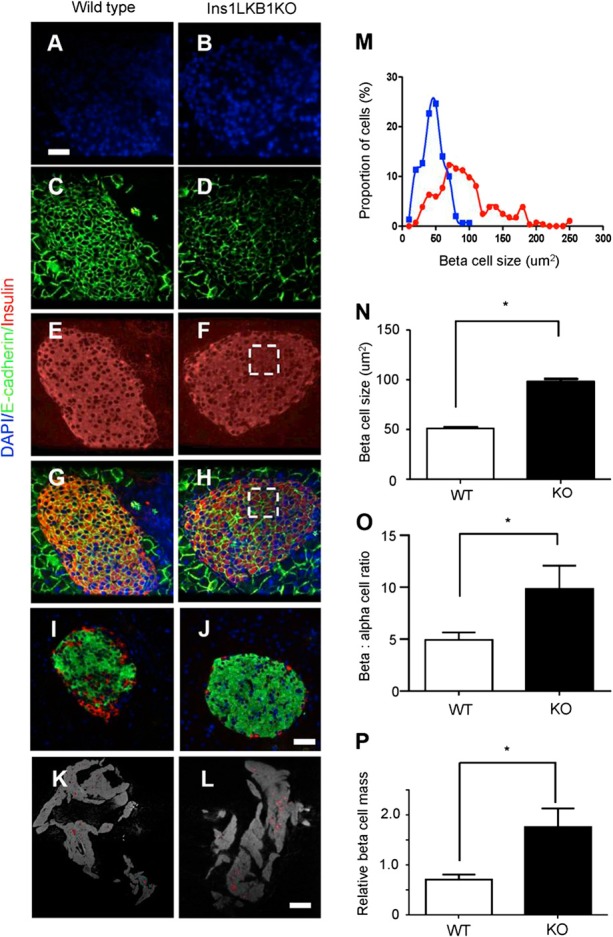
LKB1 deletion increases β-cell size and mass in Ins1LKB1KO mice. *A–J*) Consecutive pancreatic sections from WT (*A–I*) or Ins1LKB1KO (*B–J*) mice were stained for DAPI (*A*, *B*), E-cadherin (*C*, *D*), insulin (*E*, *F*), insulin plus E-cadhrin (*G*, *H*), or insulin plus GCG (*I*, *J*). Scale bars = 20 μm. *K*, *L*) Representative optical tomography projections for whole pancreata from WT (*K*) and Ins1LKB1KO (*L*) mice. Scale bar = 400 μm. *M*, *N*) Distribution of β-cell sizes (*M*) and average β-cell size (*N*) was calculated from data as shown in *C* and *D. O*) Quantitation of the data in *I* and *J. P*) Quantification of OPT data from *K* and *L. n* = 3–6 mice/genotype. *P < 0.05; Student's *t* test.

Examined at ages up to 12 wk, Ins1LKB1KO mice displayed improved glucose tolerance (**Fig. 1*A***), unaltered insulin sensitivity (Fig. 1*B*), and sharply improved *in vivo* insulin secretion (Fig. 1*C*) compared to WT littermates. An increase in β-cell size (**Fig. 2*A***–***J***), and the appearance of “rosette-like” arrangements of β cells around a central organizing center (Fig. 2*G*, *H*), were also observed, as described previously after RIP2Cre-mediated (20) or Pdx1CreER-mediated (21) LKB1 deletion. Pancreatic β-cell mass was also significantly increased (Fig. 2*K*, *L*, *P*) as well as β-cell size (Fig. 2*M*, *N*) and β:α cell ratio, consistent with earlier findings using alternative *Cre*s (20, 21). Examined *in vitro*, glucose-stimulated insulin secretion from Ins1LKB1KO islets was largely unchanged with respect to control mouse islets (Supplemental Fig. S1*A* and **Fig. 6*C***).

We next generated an analogous mouse line in which AMPK activity was eliminated selectively in the β cell by deleting both catalytic subunits of AMPK (α1 and α2). Body weight gain was not different in Ins1AMPKdKO (*Cre*^+^, α1^f/f^, α2^f/f^) mice with respect to *Cre*^−^ controls (at 10 wk, means±sem,: WT, 27.07±0.42 g; AMPKdKO, 27.75±0.20 g).

**Figure 3.**
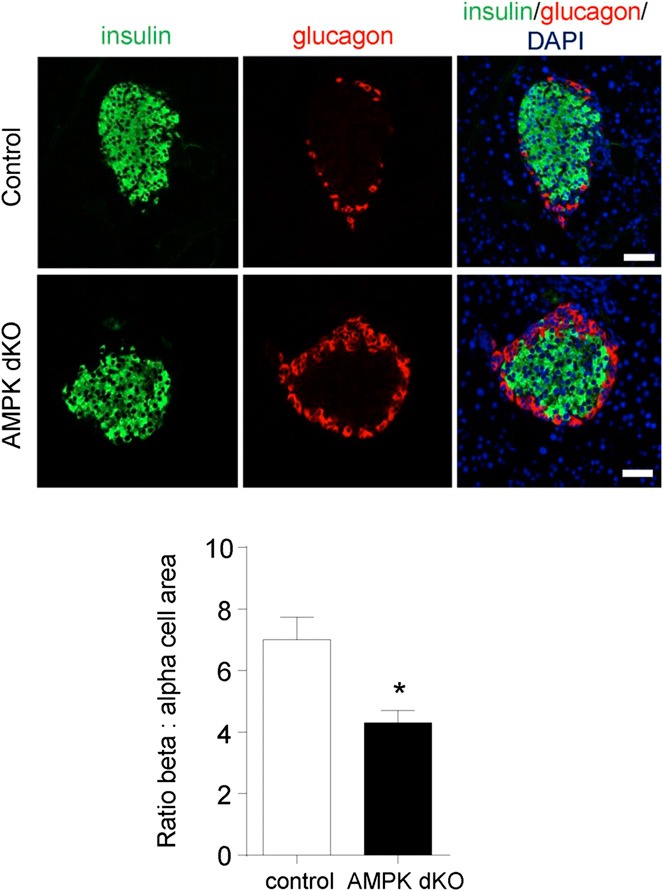
Deletion of AMPK catalytic subunits with Ins1Cre alters islet β:α cell ratio. Pancreata from Ins1AMPKdKO mice and controls were fixed, sectioned, and subjected to immunocytochemical analysis for insulin and GCG as given in Materials and Methods. *n* = 3 mice/genotype in each case. *P < 0.05; paired Student's *t* test.

Ins1AMPKdKO mice displayed a more minor phenotype compared to that observed in the RIP2AMPKdKO model (23, 24). Thus, glucose tolerance was only slightly impaired in Ins1AMPKdKO animals (Fig. 1*D*), with blood glucose significantly elevated only at 30 min during intraperitoneal glucose tests (IPGTTs). Moreover, whereas insulin sensitivity was markedly improved in RIP2AMPKdKO mice *vs.* heterozygous controls (23), no alterations in this parameter were seen in Ins1AMPKdKO animals (Fig. 1*E*). Finally, whereas the first phase of glucose-stimulated insulin release *in vivo* was completely abolished in RIP2AMPKdKO mice (23), release of the hormone was diminished by only ∼50% in Ins1AMPKdKO mice *vs.* controls (Fig. 1*F*). No evident changes in β-cell mass were observed in Ins1AMPKdKO mice *vs.* littermate controls (pancreatic β-cell area: 0.31±0.06 *vs.* 0.39±0.5%, respectively, *n*=5–6 mice/genotype), though the ratio of β:α cells was significantly reduced in these mice (**Fig. 3**). In common with RIP2AMPKdKO mice (23), a dramatic improvement in glucose-stimulated insulin secretion was observed *in vitro* with islets isolated from Ins1AMPKdKO mice (Supplemental Fig. S1*B*).

### Deep sequencing identifies gene modules differentially affected in Ins1LKB1KO and Ins1AMPKdKO mouse islets

We next explored whether the phenotypic differences between Ins1LKB1KO and Ins1AMPKdKO mouse β cells might be reflected at the transcriptome level using RNASeq (Supplemental Tables S1 and S2).

Ins1LKB1KO mouse islets displayed no significant changes in β-cell markers, including *Slc2a2 (Glut2), Pdx1, MafA, NeuroD*, and *Nkx6.2*, while *Pcsk1* mRNA was up-regulated 1.75-fold, consistent with an increase in β-cell volume within the islet. Conversely, expression of the α-cell-enriched factor *Arx* (41) was significantly (0.65-fold, *E*=0.05) lowered, and *Gcg* mRNA levels tended (0.84-fold, *E*=0.19) also to be decreased, consistent with the increase in β:α cell ratio (Fig. 2*O*). Somatostatin (*Sst*) mRNA levels also showed a minor tendency to decrease (0.74-fold, *P*=0.31) in Ins1LKB1KO islets. Significant increases were also observed in the expression of genes usually restricted to α cells (42) including *Pcsk2* [encoding prohormone convertase 2 (PC2), 1.61-fold, *E*<0.01] as well as *Dpp4* (1.51-fold, *E*=0.03). The latter findings may suggest that LKB1 ablation loosens the repression in the β cell of a subset of usually α-cell-restricted genes.

Conversely, in Ins1AMPKdKO mouse islets, *Slc2a2* (Glut2; 0.59-fold, *E*=0.04; ref. 43), mRNA levels were significantly reduced compared to WT controls, and other β-cell markers, including *Ins2* and *Slc30a8*, also tended to be more weakly expressed in the absence of AMPK. On the other hand, *Gcg* and *Arx* mRNA levels tended to increase, while *Pcsk2* was significantly up-regulated (1.21-fold, *E*<0.04). Thus, AMPK signaling is required to maintain normal islet β-cell mass and is not rescued by other AMPK-related kinases.

**Figure 4.**
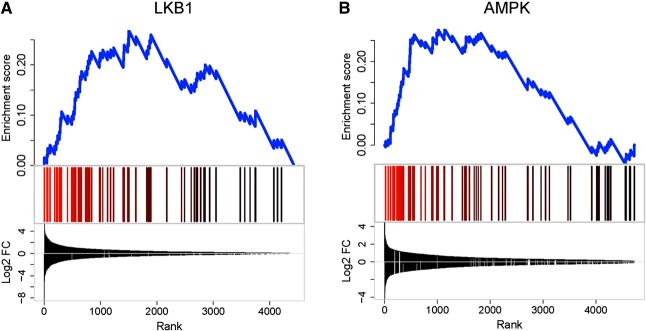
GSEA for neuronal genes in Ins1LKB1KO (*A*) or Ins1AMPKdKO (*B*) mice. *A*) GSEA was performed against MSigDB (V4) biological process gene ontolology categories (C5, BP) revealing significant enrichment for neuronal genes for Ins1LKB1KO [MSigDB category: NERVOUS_SYSTEM_DEVELOPMENT; Enrichment (ES) score=0.41 FDR=0.06]. In Ins1-AMPKdKO, this category was not significantly enriched (ES score=0.02; FDR=1; right panel). Data are expressed as absolute fold change irrespective of the direction of change. Blue line plot shows the cumulative ES score for this functional gene category. Vertical bars indicate the positions of the neuronal genes in the ranked differential expression lists. Line plot at the bottom shows the log_2_-fold changes of the genes in the ranked lists. Because the initial gene lists were ranked by absolute fold change, both up- and down-regulated genes are found at the top of the lists.

To identify gene clusters affected by either kinase, we first used GSEA (ref. 33; see Materials and Methods and Supplemental Tables S3 and S4). Whereas genes involved in neuronal function (synapse organization, synaptic transmission, synaptogenesis, *etc*.) were strongly and significantly enriched in Ins1LKB1KO islets (**Fig. 4*A***, **Table 1**, and Supplemental Fig. S2*A*), the most strongly enriched gene groups in Ins1AMPKdKO islets were involved in second messenger signaling and metal ion transport (Supplemental Table S3). Nonetheless, a tendency was observed for enrichment of neuronal genes in Ins1AMPKdKO islets, though this did not reach statistical significance (Fig. 4*B* and Supplemental Fig. S2*B*).

**Table 1.** Ins1LKB1KO and Ins1AMPKdKO: synapse cluster

Category	Term	Count	%	*P*	List total	FDR
GOTERM_CC_FAT	GO:0045202∼synapse	18	4.37	9.67E-04	284	1.24
GOTERM_CC_FAT	GO:0044456∼synapse part	14	3.40	0.00107	284	1.38
GOTERM_CC_FAT	GO:0008021∼synaptic vesicle	7	1.70	0.00430	284	5.45
GOTERM_CC_FAT	GO:0030135∼coated vesicle	8	1.94	0.0225	284	25.67
GOTERM_CC_FAT	GO:0030136∼clathrin-coated vesicle	7	1.70	0.0306	284	33.25

We next compared genes which were changed in both or in a single Ins1*Cre*-deleted model (Supplemental Tables S5–S7) and identified functional pathways using KEGG analysis (see Materials and Methods). Pathways in cancer was the top hit for genes regulated specifically in the Ins1LKB1KO model, with MAPK signaling pathway, apoptosis, and pancreatic cancer clusters all having FDR <30%. These changes are thus consistent with increased overall β-cell mass observed in Ins1LKB1KO but not observed Ins1AMPKdKO mouse islets (Supplemental Table S8).

In contrast, the gene clusters specifically enriched in Ins1AMPKdKO were topped by ribosome, with proteasome, spliceosome, and DNA replication among the top hits. This is consistent with the role of AMPK in inhibiting protein production through decreased ribosome biogenesis and protein translation, and also inhibiting proteosomal degradation of proteins in low cellular energy states (Supplemental Table S9).

Finally, we examined gene groups whose changes were common to both Ins1LKB1KO and Ins1AMPKdKO islets (Supplemental Table S10). Notable among pathways up-regulated in both models were a number of cancer and proliferation pathways, including cell cycle, melanoma, glioma, bladder cancer, and colorectal cancer. Axon guidance was also significantly enriched, suggesting that a subset of the LKB1-dependent cancer-related and neuronal genes are likely to be regulated *via* AMPK.

### Comparison between the effects of LKB1 *vs.* AMPK deletion across *Cre* models

We reasoned that comparisons of the islet transcriptome in additional β-cell-selective LKB1KO and AMPKKO models might validate targets for each kinase identified above in the Ins1*Cre*-deleted models and provide further evidence for β-cell-autonomous actions of each kinase. We therefore performed deep sequencing on islets from mice inactivated for either LKB1 (20) or AMPK (23) using the RIP2Cre transgene, as described previously (Supplemental Tables S11 and S12). In addition, microarray analysis was performed on islets from Pdx1CreERLKB1KO mice (ref. 21 and Supplemental Table S13). In the latter model, LKB1 deletion was achieved acutely in the adult mouse β cell by tamoxifen-mediated activation of *Cre* (21).

**Figure 5.**
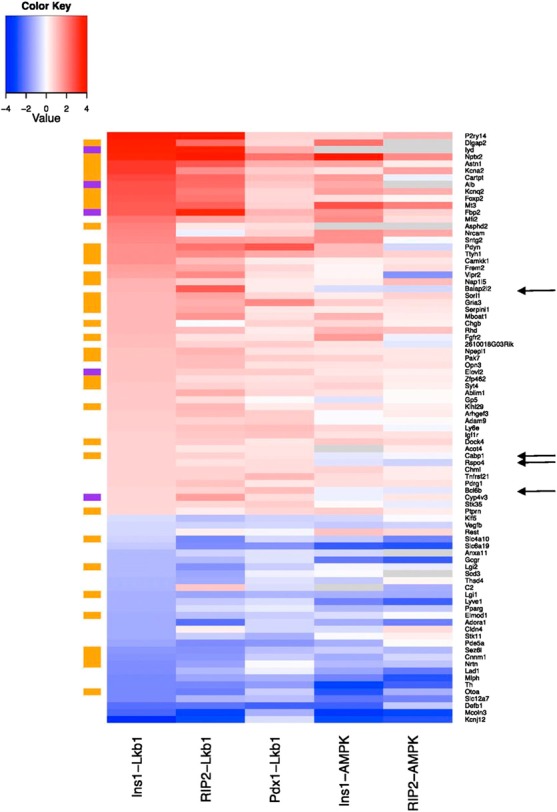
Differentially regulated genes after LKB1 or AMPK deletion in β cells. Heat map showing genes significantly up-regulated (red hues) or down-regulated (blue hues) by >1.4-fold in islets after deletion of the indicated kinases. Gray indicates genes absent from 1 or more samples (see Supplemental Tables S1 and S2). All genes shown were up-regulated in Ins1CreLKB1KO *vs.* control islets and ≥1 other LKB1KO model. Color bars at left indicate neuronal genes (brown) and liver-enriched genes (purple). Arrows indicate messages selectively enriched in LKB1 *vs.* AMPKdKO models.

We next ranked the changes in mRNAs levels in Ins1LKB1KO *vs.* control islets. Genes were further filtered as displaying a significant (*E*<0.1 or *P*<0.05) and ≥1.4-fold change in at least one further LKB1-null strain (RIP2Cre or Pdx1CreER). We note that this conservative approach may conceivably exclude genuine β-cell targets, should these be further modified as a result of undefined brain-derived signals, but is likely to be relatively less prone to identifying false positives (*i.e.*, a type I statistical error). A comparison of the changes observed was then made for each gene in the 2 AMPKdKO models. The resulting analysis is shown in **Fig. 5** and Supplemental Fig. S3. A clear conservation of differentially expressed genes was apparent when considering as groups the 3 LKB1-null and the 2 AMPK-null models, irrespective of the *Cre* used (Supplemental Fig. S3). More divergence was seen between the models deleted for either kinase, as anticipated. Thus, all 12 of the most induced genes in Ins1LKB1KO mouse islets were also up-regulated in RIP2LKB1KO and Pdx1CreERLKB1KO islets (Fig. 5). Of these, 8/12 and 7/12, respectively, were also increased in Ins1AMPKdKO and RIP2AMPKdKO mice (Fig. 5). Moreover, 4/12 (*Nptx2*, *Astn1*, *Kcnq2*, and *Mt3*) were significantly up-regulated in all 5 KO models, suggesting that they represent biologically relevant targets for LKB1-AMPK signaling in the β cell.

Strikingly, 49% (27/55) of the most up-regulated genes in Ins1LKB1KO islets, and one other LKB1-null strain, were usually expressed exclusively or principally in neuronal tissues (as identified using BioGPS); this proportion rose to 56% for the 25 most highly induced genes. A further 9.1% (5/55) were genes enriched in the liver or small intestine. These proportions were 40 and 11%, respectively, for the top 45 differentially expressed genes. Of the top 12 most strongly induced genes in Ins1LKB1KO mouse islets, 10 fell into one of these 2 groups (Fig. 5). The degree of overlap between different models is shown in Supplemental Figs. S6–S8.

Divergence was less marked for down-regulated genes, with the expression of all 11 of the most-repressed genes in Ins1LKB1KO islets also being similarly inhibited in the 4 other KO models, albeit with differences in rank order (Fig. 5). A number (8/30, 26.6%) of neuronally enriched genes were also present in the repressed group. Interestingly, 2 of the 4 most repressed genes across all 5 KOs, *Slc12a7*, encoding a K/Cl^−^ symporter, and *Mcoln3* (also called Trpm3), are usually very weakly expressed in neuronal tissues, but strongly expressed in other tissues.

### Glutamate signaling and addiction-related genes

**Figure 6.**
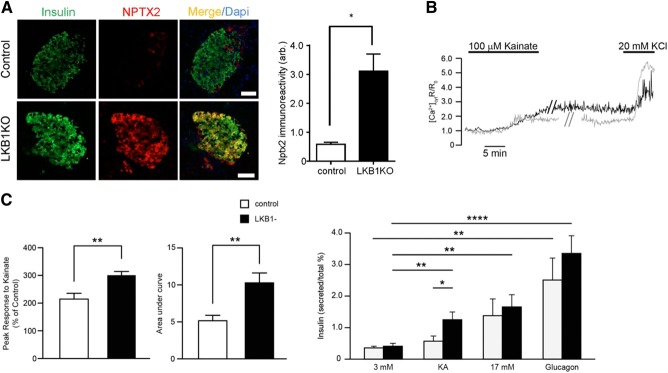
Up-regulation of neuronal genes in Ins1LKB1KO mice is associated with enhanced glutamate signaling. *A*) Immuncytochemical analysis of pancreatic slices from the indicated mouse strains stained for NPTX2. Scale bar = 50 μm. *B*) Glutamate receptor signaling to increases in intracellular [Ca^2+^]_cyt_ in control (gray trace; *n*=18) and Ins1LKB1KO (black; *n*=16) separate β cells, from 6 independent experiments. Mean amplitude of [Ca^2+^] peak and AUC in response to kainite were gathered from 7 WT and 6 Ins1LKB1KO mice. **P < 0.01. *C*) Insulin secretion in response to kainate and high glucose in islets from control and Ins1LKB1KO mice (*n*=4 animals/genotype). Incubations were performed in triplicate and involved 6 islets/tube. *P < 0.05, **P < 0.01, ****P < 0.0001.

More detailed inspection identified important functional clusters among the neuronally enriched genes (Fig. 5; Supplemental Fig. S3). First, a subset was identified that was involved in glutamate signaling (*Nptx2, Gria3, Dlgap*2), while 2 genes (*Cartpt* and *Pdyn*) are usually associated with addictive behavior. Up-regulation of the former group was apparent in all 3 LKB1KO models, while *Gria1*, encoding the ionotropic glutamate receptor GluR1 (44), was also up-regulated in Ins1LKB1KO islets. *Nptx2* and *Dlgap2* expression was also significantly increased in Ins1AMPKdKO islets (Supplemental Table S2). *Dlgap2* encodes discs, large (Drosophila) homologue-associated protein 2 (45), a membrane-associated protein that forms part of the postsynaptic density scaffold and interacts with postsynaptic density 95 (PSD95). The latter, in turn, binds to *N*-methyl-d-aspartate (NMDA) receptors and Shaker-type K^+^ channels. Interestingly, *Kcna2*, a Shaker channel, was also strongly up-regulated by LKB1 or AMPK deletion (Fig. 5). Immunocytochemistry confirmed the induction of NPTX2 at the protein level in Ins1LKB1KO β cells (Fig. 6*A*).

Consistent with increased glutaminergic signaling in Ins1LKB1KO β cells, kainate, which binds to and activates ionotropic glutamate receptors (44), caused a more pronounced rise in cytosolic-free Ca^2+^ ([Ca^2+^]_cyt_) in islets from Ins1LKB1KO than control mice (Fig. 6*B*). Furthermore, at low (3 mM) glucose, kainite stimulated insulin secretion from Ins1LKB1KO but not control islets (Fig. 6*C*).

### Disallowed genes

**Figure 7.**
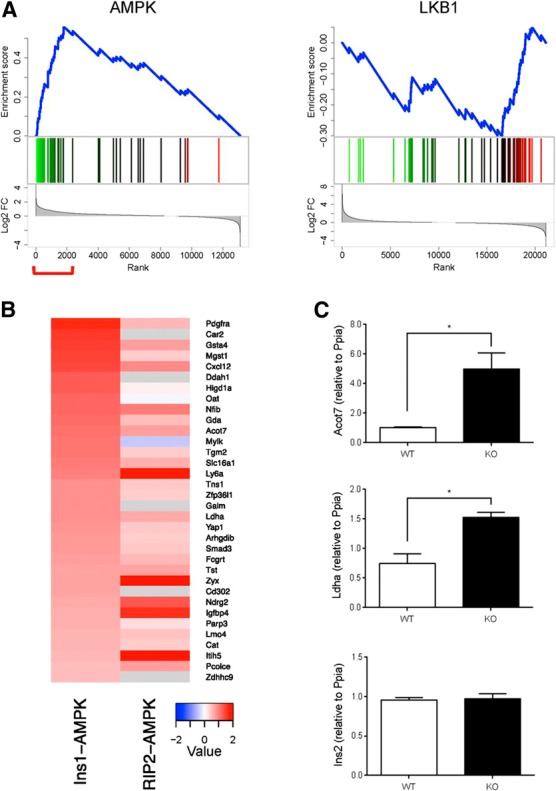
GSEA for disallowed genes in Ins1LKB1KO and Ins1AMPKdKO mouse islets. *A*) Analyses were performed as described in Materials and Methods and in the legend to Fig. 4. Disallowed genes were selected as described previously (9, 10, 46). Here the differentially expressed genes were ranked by fold change (high to low) and tested for enrichment against the set of disallowed genes. Genes with increased expression are indicated in green, decreased expression in red. Plot at bottom indicates log_2_-fold changes of the genes. Ins1AMPKdKO and RIP2AMPKdKO show enrichment for disallowed genes among the genes that are up-regulated (left panel). In contrast, the situation with LKB1 is less clear, with Ins1LKB1KO showing enrichment for disallowed genes among the down-regulated genes (top right), and RIP2LKB1KO showing no clear enrichment for disallowed genes (bottom right). Red bracket indicates significantly enriched genes. *B*) Comparison of up-regulated disallowed genes in Ins1AMPKdKO and RIP2AMPKdKO mice. Heat maps show the ranking of disallowed gene changes. *C*) *Acot7* and *Ldha* expression is up-regulated in β cells lacking AMPKα1/α2. Expression of *Ins2, Acot7*, and *Ldha* was measured by qRT-PCR in β cells sorted from islets corresponding to control and Ins1AMPKdKO mice (*n*=3 animals/genotype). The following specific primers were used: *Ins2*, 5′-CGTGGCTTCTTCTACACACCC-3′ and 5′-AGCTCCAGTTGTGCCACTTGT-3′, *Acot7*, 5′-TCTTCACCTACGTGTCCCTGAA-3′ and 5′-TCCGTCTCTGGCACAAGCT-3′ and *Ldha*, 5′-ATGAAGGACTTGGCGGATGA-3′ and 5′-ATCTCGCCCTTGAGTTTGTCTT-3′.Values were normalized to Ppia and expressed in relative quantities related to a control mouse. Data are shown as means ± sem. *P < 0.05.

We (9, 10) and others (46) have previously described a list of >60 housekeeping genes that are expressed in the majority of mammalian tissues at quite high levels, but at much lower levels in islets and β cells; of these, 11 genes were reported in both of the previous studies (10). We noticed that several of the genes in both lists were up-regulated in RIP2AMPKdKO and Ins1AMPKdKO islets. GSEA analysis (**Fig. 7*A***) revealed that 16 members of this family were specifically up-regulated in Ins1AMPKdKO islet models. By contrast, Ins1CreLKB1KO islets showed a negative enrichment for this class (Fig. 7*A*).

The most up-regulated genes in this group are compared in the Ins1AMPKdKO and RIP2AMPKdKO models in Fig. 7*B*. Suggesting that these increases are unlikely to reflect an enrichment of AMPKdKO islets with nonislet material, levels of the pancreatic acinar cell marker amylase were not different between Ins1- or RIP2AMPKdKO *vs.* control islets. Moreover, examined in FACS-sorted mouse islet cells (47), with the exception of *Oat* and *Igfbp4*, similar levels of expression of each gene were detected in WT β and α cells (Supplemental Table 14). Finally, and providing direct confirmation of the above conclusion, expression of both *Ldha* and *Acot7* were significantly increased in FACS-purified β cells from Ins1AMPKdKO *vs.* control mice (Fig. 7*C*).

### Hepatic genes

Striking changes were observed across all 3 LKB1KO plus Ins1AMPKdKO models in the expression of 3 genes largely restricted to the liver, namely, albumin (*Alb*), iodotyrosine deiodinase (*Iyd*), and elongation of very long chain fatty acids 2 (*Elovl2*). Furthermore, the liver/kidney-enriched gluconeogenic gene, fructose 1,6-bisphoshatase-1 (*Fbp1*), was significantly up-regulated in 2 of the 3 LKB1KO models (RIP2- and Pdx1CreER), and tended (*P*=0.12) to be activated in Ins1LKB1KO mouse islets. Fructose 1,6-biosphosphatase-2 (*Fbp2*), usually restricted to stomach, small intestine, and skeletal muscle, was significantly up-regulated in each of the LKB1 models and in RIP2AMPKdKO mice (Fig. 5), suggesting increased potential for gluconeogenesis.

### G-protein-coupled receptors

Purinergic receptor P2Y, G-protein coupled, 14 (*P2ry14*) expression was the most strongly (∼16-fold) up-regulated gene in Ins1LKB1KO and RIP2LKB1KO islets and was also induced in Pdx1CreERLKB1KO and both AMPKdKO models (Fig. 5).

Interestingly, GCG receptor (*Gcgr*) expression was substantially (∼2-fold) reduced after LKB1 deletion in all 3 models, and even more markedly (∼4-fold) reduced in Ins1AMPKdKO islets, with also a strong tendency (*P*=0.064) toward a reduction in RIP2AMPKdKO islets. Thus, *Gcgr* seems likely to be a direct target for the actions of AMPK. Similarly, glucose-dependent insulinotropic polypeptide (GIP) receptor (*Gipr*) and GCG-like peptide-1 (GLP-1) receptor (*Glpr*) expression were both significantly (∼50%; *P*<0.002, 0.007, respectively) down-regulated in Ins1AMPKdKO islets.

### T2D-associated genes

Genome-wide association studies (GWASs) have now identified >70 loci associated with T2D (48). Of the genes closest to the implicated single-nucleotide polymorphisms (SNPs), we identified relatively few whose expression was affected in any of the LKB1KO or AMPKKO models. However, the GWAS gene transducin-like enhancer of split 1 (*Tle1*), encoding an RNA pol II interactor involved in Wnt and Notch signaling, as well as histone deacetylase recruitment (49), was significantly up-regulated in all 3 LKB1KO models examined and in Ins1AMPKdKO islets. Furthermore, expression of *Dock4*, encoding the guanine nucleotide exchange factor for Rac, and another binding partner of β-catenin and regulator of Wnt signaling (50), was up-regulated in all LKB1KO models and in Ins1AMPKdKO islets (Fig. 5). These observations support earlier findings (51) indicating that LKB1, at least partly *via* AMPK activation, suppresses canonical Wnt signaling in mammalian cells.

### T2D-regulated genes

We next asked whether a decrease in LKB1 or AMPK activity in the islet might lead to changes that mimic those observed in T2D (52). Implicating a role for impaired AMPK activation in the disease, the β-cell-disallowed genes *Ldha, Slc16a1, Acot7, Mgst1*, and Pdgfra are all increased T2D islets (52). On the other hand, *Nptx2, Dlgap2*, and Cartpt are not reported to change.

### Common promoter regions in the regulated genes

We wondered whether the LKB1/AMPK-regulated genes may be the target for a common set of transcription factors. To this end, we searched for conserved TFBSs overrepresented in the upstream regions of differentially expressed genes, as described in Materials and Methods. This revealed sites common to a range of factors, as shown in **Table 2**. Strikingly, the most enriched TFBS upstream of genes differentially expressed in both Ins1LKB1KO and Ins1AMPKdKO was Zfp206 (*Znf206*/*Zscan10*) (*P*=1.3×10^−33^). Increased expression of this gene is associated with neuronal differentiation (53), and several of the most up-regulated neuronal genes (*e.g.*, *Nptx2* and *Dlgap2*) have putative Zfp206 binding sites. Zfp206 is more significantly enriched in genes only differentially expressed in the Ins1LKB1KO model (*P*=9.1×10^−96^), consistent with the more significant degree of up-regulation of neuronal genes observed in this model *vs.* Ins1AMPKdKO (Fig. 4 and Supplemental Figs. S3 and S4).

**Table 2.** Top 10 transcription factor binding sites enriched upstream of genes differentially expressed in Ins1LKB1KO and Ins1AMPKdKO

Rank	Name	Hits in submitted regions	Hits on genome	−log_10_ (*P*)
1	Zfp206	1997	23,846	32.8816
2	E2F-4	2040	25,565	23.3829
3	SP1:SP3	2445	31,775	20.0247
4	Egr-1	2649	35,444	15.8367
5	HIF1	612	6,863	15.6096
6	SAP-1a	1322	16,794	13.8834
7	SP2	1380	17,684	13.3895
8	GABPalpha	2155	28,850	12.9993
9	GABP	2693	36,795	12.4291
10	SP4	1415	18,330	12.4105

Hypoxia-inducible factor 1 (HIF1) binding sites were also enriched in genes differentially expressed in both models (*P*=2.5×10^−16^). More detailed examination, taking the direction of differential expression into account, revealed that HIF1 sites were enriched in genes significantly up-regulated in just the Ins1AMPKdKO model (*P*=5.7×10^−13^) and also in genes down-regulated in just the Ins1LKB1KO model (*P*=2.2×10^−26^). LKB1 and AMPK therefore both appear to have nonoverlapping roles in HIF1 regulation. Hypoxia is known to up-regulate a number of β-cell disallowed genes (*e.g.*, *Ldha* and *Slc16a1*; ref. 54), and the enrichment of HIF1 binding sites in genes selectively up-regulated in Ins1AMPKdKO supports a role for this transcription factor in disallowed gene suppression.

## DISCUSSION

The present study provides the first detailed transcriptomic analysis exploring the roles of LKB1 and AMPK in the endocrine pancreas. First, we confirm several previous findings (20–23) based on the deletion of these kinases in multiple tissues, showing that when restricted to the β cell, loss of LKB1 exerts markedly different effects on insulin secretion and glucose metabolism compared to the loss of AMPK (Figs. 1–3). We now demonstrate that these functional differences are mirrored by complex alterations at the level of gene expression (Figs. 4 and 5, Supplemental Figs. S1–S3, and Supplemental Tables S1–S13).

Differences in gene expression between Ins1LKB1KO and Ins1AMPKdKO islets are likely to reflect signaling by members of the AMPKRK family (16) in the former case. However, the identity of the kinases involved is unclear. Thus, deletion of synapses of amphids defective kinase-A (SAD-A) in the pancreas causes *defective* glucose signaling and the appearance of small islets (55), suggesting that this enzyme rather opposes the actions of LKB1, mediating instead the effects of mammalian target of rapamycin-1 (mTORC1). Likewise, deletion of salt-inducible kinase 2 (SIK2; ref. 56) impairs glucose tolerance and insulin secretion.

An interesting observation, replicating findings in RIP2AMPKdKO islets (23), is that while glucose-stimulated insulin secretion was strongly impaired *in vivo* in Ins1AMPKdKO mice (Fig. 1*C*), it was enhanced from isolated islets (Supplemental Fig. 2*B*). There may be several explanations for this difference. First, it may reflect the loss *in vitro* of an action of circulating factors that normally regulate insulin secretion *in vivo*. Obvious candidates are the incretins GIP and GLP-1, as well as GCG, given the lowered expression of the corresponding receptors in Ins1AMPKdKO mice. In addition, we noted a significant (*P*<0.01) ∼2-fold up-regulation in the expression of both the somatostatin receptor gene *Sstr1*, and the adrenoreceptor *Adrb1* in Ins1AMPKdKO islets, expected to potentiate the inhibitory actions of the cognate hormones on insulin secretion; neither gene was affected in Ins1LKB1KO islets.

### Identification of genes differentially regulated by LKB1 and AMPK

It seems reasonable to speculate that genes affected by the loss of LKB1, but not AMPK, may contribute to enhanced insulin secretion in the former. Of the genes most strongly up-regulated in all 3 LKB1KO models, 4 were unaffected in both AMPKdKO models: *Baiap2l2, Cabp1, Rspo4*, and *Bcl6b* (Fig. 5). *Bcl6b* encodes a transcription factor and downstream target of FGF2 capable of promoting germ cell tumors (57). Likewise, RSPO4 is a member of the R-spondin family of secreted agonists of canonical Wnt/β-catenin signaling that binds to Lgr receptors to enhance cell growth (58). BAIAP2L2 is a member of the I-BAR domain family of proteins involved in the control of actin dynamics and signaling at glutaminergic synapses (59) and filopodia extension (60), and may thus also be involved in Wnt or Hedgehog signaling. Finally, CaBP1 is a neuronal calcium binding protein that regulates several Ca^2+^ channels, including transient receptor potential 5 (Trp5; ref. 61).

### Regulation of neuronal genes in the β cell by LKB1 and AMPK

A striking finding of the present study is that both LKB1 and AMPK control the expression of a large number of neuronal genes in the β cell. LKB1 deletion had the most dramatic effect on this group of genes (Fig. 4*A*), and this effect was also observed after short-term (1 wk) loss of LKB1 in the Pdx1CreER-deleted model. Several of the most strongly affected genes, notably *Nptx2* and *Dlgap2*, were also subject to control by AMPK (Fig. 5). Thus, both AMPK and AMPKRKs may mediate the effects of LKB1 on this gene group. Analysis of the promoter regions of genes regulated by either kinase revealed several potential downstream regulators (Table 2), of which Zfp206, a neuron-enriched transcription factor, is an attractive candidate for mediating these effects.

We also considered the possibility that some of the changes may be the result of altered expression of repressor element 1 silencing transcription factor (REST), which represses neuronal genes in non-neuronal cells and is also relatively weakly expressed in unmodified adult β cells (62). However, while REST expression was reduced in islets from Ins1LKB1KO islets, its expression was slightly increased, or barely affected, in RIP2LKB1KO and Pdx1CreERLKB1KO islets, respectively, while REST expression was slightly *increased* in both AMPKdKO model islets (Fig. 5). Nonetheless, differences in REST expression between Ins1LKB1 and the AMPKdKOs may contribute to the differential enrichment for neuronal genes (Fig. 4). Thus, several well-defined targets for REST, including *Snap25* (*P*=0.05) and the synaptotagmin family member *Syt4* (*P*<0.01), were up-regulated in Ins1LKB1KO islets.

### Regulation in the β cell of genes involved in glutamate signaling and addictive behavior

*Gria3*, encoding the GluR3 glutamate receptor, was up-regulated in all 5 KO models, while *Gria1* (GluR1) expression was also increased in Ins1LKB1KO islets, as were *Dlgap2* and *Nptx2. Nptx2* encodes neuronal pentraxin 2 (also known as Narp in the rat), which binds to the extracellular domain of α-amino-3-hydroxy-5-methyl-4-isoxazolepropionic (AMPA)-activated subclass of glutamate receptors, causing their clustering. Thus, pentraxins are secreted from presynaptic neurons (63), and recruit GluR4 AMPA receptors to synapses. Pentraxins are also implicated in neuronal plasticity and the behavioral responses to drug abuse (64).

We show that induction of *Nptx2* (alongside that of other genes involved in glutamate signaling; see above) was accompanied functionally by enhanced kainite-induced Ca^2+^ signals, and insulin secretion (Fig. 6*B*, *C*). This suggests that LKB1, and possibly AMPK, are necessary to suppress pathways that may lead to the dysregulation of secretion in β cells.

### Regulation of disallowed genes by AMPK

Our finding here that loss of AMPK (but not LKB1) from β cells up-regulates the β-cell-disallowed gene family (10) raises the interesting possibility that this enzyme is a key determinant in maintaining normal glucose responsiveness. Thus, elevated AMPK activity in the β cell between meals is likely to be required to maintain low levels of these enzymes. Indeed, AMPK activators such as metformin, used clinically as oral antihyperglycemics, may act in part in this way (18).

We have considered the possibility that an increase in postprandial glycemia in the *AMPKdKO* mouse *vs.* controls may contribute to the up-regulation of these genes (6, 65). However, and against this view, neither fasting (Fig. 1*D*) nor fed (10.9±0.26 and 10.8±0.2 mM, for WT and Ins1AMPKdKO mice, respectively; *n*=4, *P*>0.05) glycemia differed between genotypes; and mRNA changes in Ins1AMPKdKO islets were preserved after culture at 11 mM glucose for 24 h postisolation.

## CONCLUSIONS

LKB1 and AMPK are shown here to be powerful regulators of β-cell differentiation, acting partly in concert and partly independently of one another. Although cell fate switching is implied by the effects of loss-of-function mutations in LKB1 in Peutz-Jeghers syndrome (19), to our knowledge this report provides the first example of developmental fate being affected by the fuel-sensitive protein kinase AMPK. Interestingly, these changes occurred without apparent reversion to a more progenitor-like state, as indicated by unchanged expression of *Ngn3*, *Oct4*, or *Nanog*, in contrast to recently described models (8).

Further dissection of the mechanisms acting downstream of LKB1 in β cells may thus provide the basis of strategies to maintain cellular differentiation in some forms of diabetes.

## Supplementary Material

Supplemental Data
