# Supplementary material for: LKB1 and AMPK differentially regulate pancreatic β-cell identity
Source: FASEB J. 2014 Nov;28(11):4972–85. doi: 10.1096/fj.14-257667 (PMC4377859; doi:10.1096/fj.14-257667)
Supplement: Supplemental Data [file supp_28_11_4972__index.html]

LKB1 and AMPK differentially regulate pancreatic β-cell identity — LKB1 and AMPK differentially regulate pancreatic β-cell identity — Supplemental Data 

# LKB1 and AMPK differentially regulate pancreatic β-cell identity

## Supplemental Data

**Files in this Data Supplement:**

- Supplemental Data - (*14-257667SuppData.zip; compressed file 7.17 MB*)
