# Supplementary figures and images for: LKB1 and AMPK differentially regulate pancreatic β-cell identity
Source: FASEB J. 2014 Nov;28(11):4972–85. doi: 10.1096/fj.14-257667 (PMC4377859; doi:10.1096/fj.14-257667)

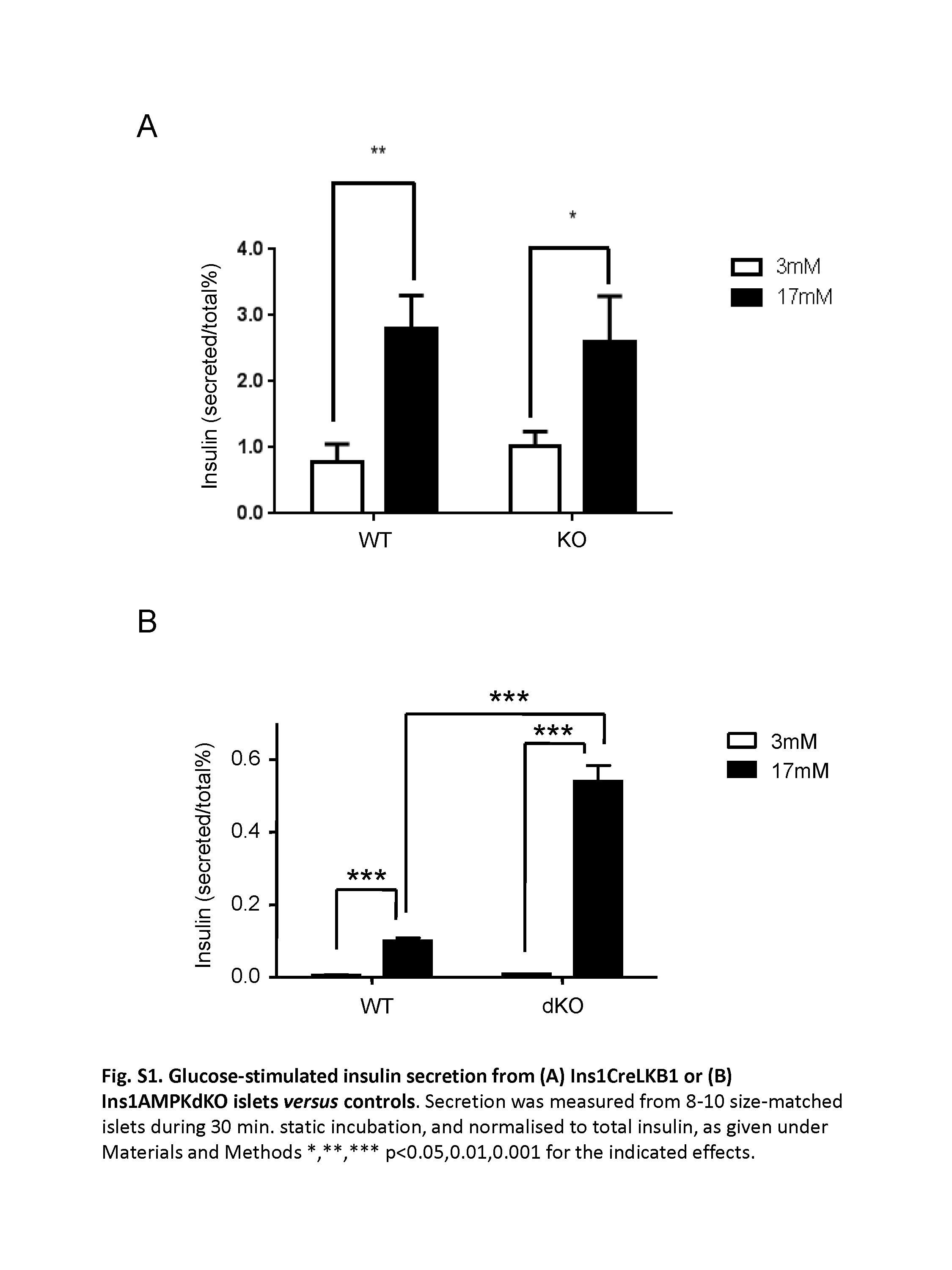

Supplement: Supplemental Data [file supp_fj.14-257667_14-257667SuppData.zip › Figures_080614_FASEBJ_V2-1_Page_08.tif]

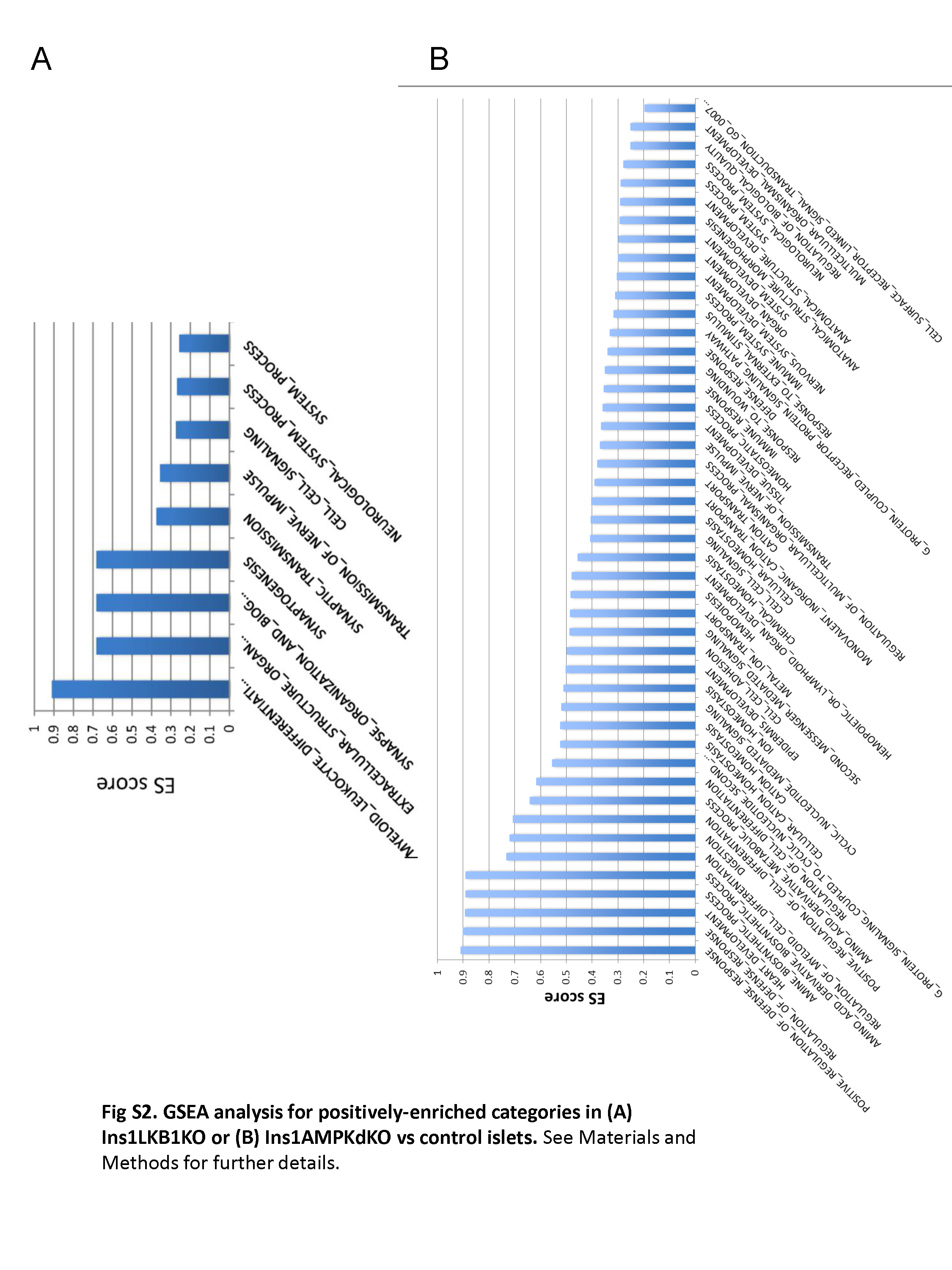

Supplement: Supplemental Data [file supp_fj.14-257667_14-257667SuppData.zip › Figures_080614_FASEBJ_V2-1_Page_09.tif]

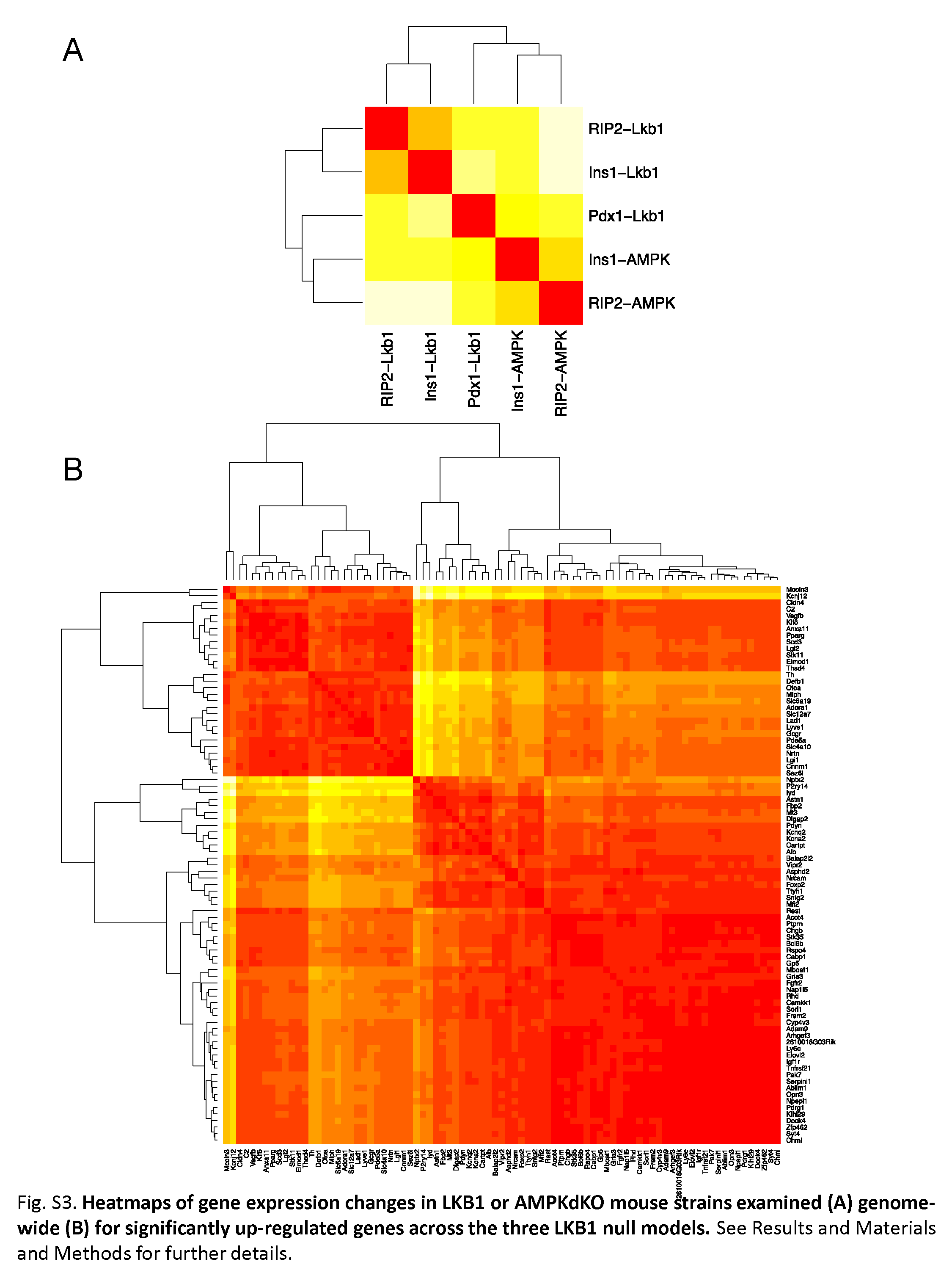

Supplement: Supplemental Data [file supp_fj.14-257667_14-257667SuppData.zip › Figures_080614_FASEBJ_V2-1_Page_10.tif]
